# HDAC8 and STAT3 repress *BMF* gene activity in colon cancer cells

**DOI:** 10.1038/cddis.2014.422

**Published:** 2014-10-16

**Authors:** Y Kang, H Nian, P Rajendran, E Kim, W M Dashwood, J T Pinto, L A Boardman, S N Thibodeau, P J Limburg, C V Löhr, W H Bisson, D E Williams, E Ho, R H Dashwood

**Affiliations:** 1 Linus Pauling Institute, Oregon State University, Corvallis, OR, USA; 2 Department of Biostatistics, Vanderbilt University School of Medicine, Nashville, TN, USA; 3 Center for Epigenetics & Disease Prevention, Institute of Biosciences & Technology, Texas A&M Health Science Center, Houston, TX, USA; 4 Department of Biochemistry & Molecular Biology, New York Medical College, Valhalla, NY, USA; 5 Mayo Clinic, Rochester, MN, USA; 6 College of Veterinary Medicine, Oregon State University, Corvallis, OR, USA; 7 Department of Environmental and Molecular Toxicology, Oregon State University, Corvallis, OR, USA; 8 Department of Nutrition & Food Science, Texas A&M University, College Station, TX, USA; 9 Department of Clinical Cancer Prevention, MD Anderson Cancer Center, Houston, TX, USA; 10 Department of Molecular & Cellular Medicine, Texas A&M University College of Medicine, College Station, TX, USA

## Abstract

Histone deacetylase (HDAC) inhibitors are undergoing clinical trials as anticancer agents, but some exhibit resistance mechanisms linked to anti-apoptotic Bcl-2 functions, such as BH3-only protein silencing. HDAC inhibitors that reactivate BH3-only family members might offer an improved therapeutic approach. We show here that a novel seleno-*α*-keto acid triggers global histone acetylation in human colon cancer cells and activates apoptosis in a p21-independent manner. Profiling of multiple survival factors identified a critical role for the BH3-only member Bcl-2-modifying factor (Bmf). On the corresponding *BMF* gene promoter, loss of HDAC8 was associated with signal transducer and activator of transcription 3 (STAT3)/specificity protein 3 (Sp3) transcription factor exchange and recruitment of p300. Treatment with a p300 inhibitor or transient overexpression of exogenous HDAC8 interfered with *BMF* induction, whereas RNAi-mediated silencing of STAT3 activated the target gene. This is the first report to identify a direct target gene of HDAC8 repression, namely, *BMF*. Interestingly, the repressive role of HDAC8 could be uncoupled from HDAC1 to trigger Bmf-mediated apoptosis. These findings have implications for the development of HDAC8-selective inhibitors as therapeutic agents, beyond the reported involvement of HDAC8 in childhood malignancy.

Histone deacetylase (HDAC) enzymes have been implicated in both normal physiology and pathophysiology, and several HDAC inhibitors have entered clinical trials.^1, 2, 3^ These inhibitors include vorinostat, a direct-acting compound that fits the HDAC active site, and romidepsin, a prodrug that through reductive metabolism generates a zinc-binding thiol.^4^ Evaluation of such compounds against solid tumors and hematological malignancies has provided insights into clinical efficacy, as well as resistance mechanisms and off-target effects.^1, 2, 3^

Off-target effects such as cardiotoxicity and thrombocytopenia arise from inhibitors acting on multiple HDACs,^5,6^ whereas resistance has been linked to anti-apoptotic Bcl-2 functions, including BH3-only protein silencing.^7, 8, 9^ HDAC inhibitors that reactivate BH3-only family members, such as Bcl-2-modifying factor (Bmf), might offer an improved therapeutic approach for treating cancer and other conditions.

We previously reported on the HDAC inhibitory activity of methylselenopyruvate (MSP), a novel seleno-*α*-keto acid metabolite generated by transamination of the natural organoselenium compound methylselenocysteine (MSC).^10, 11, 12^ MSP was identified as a competitive inhibitor of HDAC8, and this was supported by molecular modeling studies of MSP/HDAC8 interactions *in silico*.^10^

HDAC8 was the first human HDAC to be crystalized with bound inhibitors,^13, 14, 15, 16^ but it remains one of the least understood HDACs, with no known direct transcriptional target or corepressor partner.^17,18^ In pursuing the mechanism of action of MSP in human colon cancer cells, we identified a key role for HDAC8 in regulating *BMF* gene activity and Bmf-mediated apoptosis. The work provides support for HDAC8 as a relevant mechanistic target for cancer therapy, beyond its reported involvement in childhood cancer.^19^

## Results

### MSP triggers Bmf-mediated apoptosis independent of p21 induction

**Figure 1:**
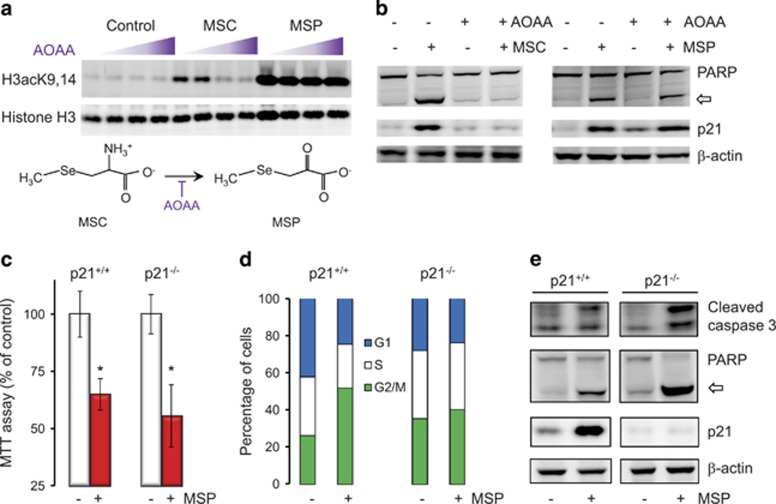
Induction of p21 by MSP is not a prerequisite for apoptosis in colon cancer cells. (**a**) HCT116 cells were incubated with the transaminase inhibitor aminooxyacetic acid (AOAA) 1 h before treatment with MSP, MSC, or vehicle (control). Whole-cell lysates were immunoblotted 6 h later for histone H3 acetylation. Wedge symbol indicates increasing concentration of AOAA. (**b**) AOAA pretreatment for 1 h blocked p21 induction by MSC and the cleavage of PARP, whereas MSP was unaffected by AOAA. Open arrow, cleaved PARP. (**c**) HCT116^p21+/+^ and HCT116^p21−/−^ cells responded similarly to MSP treatment in MTT assays; mean±S.D., *n*=3, *P<0.05 using Students *t*-test. (**d**) G_2_/M cell cycle arrest at 48 h required the presence of p21. (**e**) In the absence of p21, MSP strongly increased cleaved caspase-3 and (open arrow) cleaved PARP. Results in **a–e** are representative findings from two or more independent experiments

Metabolic conversion of MSC to MSP was reported previously.^11^ An inhibitor of aminotransfer reactions caused a dose-dependent loss of histone acetylation in human colon cancer cells treated with MSC, but not MSP (Figure 1a). This was associated with loss of cleaved poly(ADP-ribose)polymerase (PARP) and p21 induction by MSC, whereas MSP was effective in the presence or absence of the transaminase inhibitor (Figure 1b). HCT116^p21+/+^ and HCT116^p21−/−^ cells treated with MSP exhibited similar responses in the MTT (3-(4,5-dimethylthiazol-2-yl)-2,5-diphenyltetrazolium bromide) assay (Figure 1c), but p21^−/−^ cells did not accumulate in the G_2_/M phase of the cell cycle, unlike p21^+/+^ cells (Figure 1d). Notably, HCT116^p21−/−^ cells had higher levels of cleaved caspase-3 and cleaved PARP compared with HCT116^p21+/+^ cells exposed to MSP under the same conditions (Figure 1e). These findings suggested that p21 induction is necessary for G_2_/M arrest, but is not a prerequisite for apoptosis induction by MSP.

**Figure 2:**
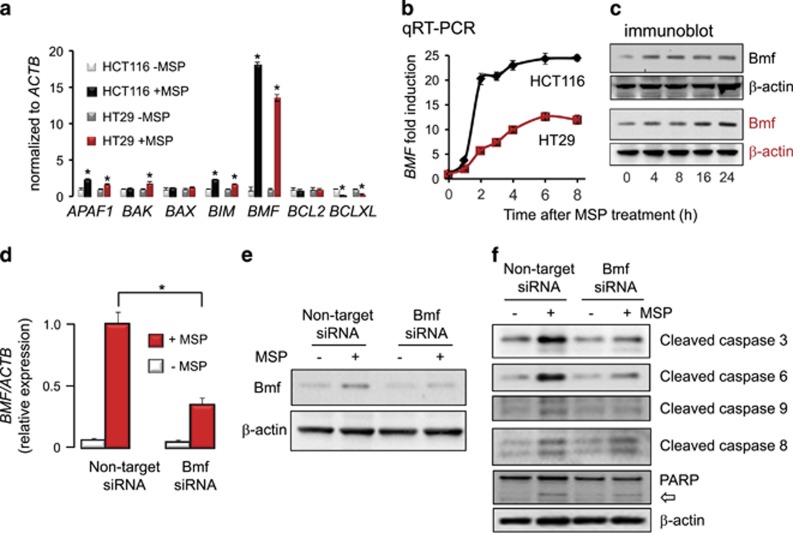
Bmf has a pivotal role in the apoptotic mechanism triggered by MSP. (**a**) Quantitative real-time PCR (qRT-PCR) assays of survival genes normalized to *β*-actin *(ACTB*), 12 h after treatment of colon cancer cells with MSP (10 *μ*M) or vehicle. Data indicate mean±S.D., *n*=3, *P<0.05 using Students *t*-test to compare MSP-treated cells with vehicle controls. (**b**, **c**) Time-course for induction of *BMF* mRNA (mean±S.D., *n*=3) and Bmf protein expression. (**d**, **e**) In HCT116 cells, MSP was significantly less effective at inducing *BMF* mRNA (mean±S.D., *n*=3, *P<0.05) and Bmf protein expression following siRNA-mediated knockdown of Bmf. (**f**) Reduced cleavage of caspases and (open arrow) PARP in MSP-treated HCT116 cells following Bmf knockdown. Results in **a–f** are representative findings from two or more experiments

To investigate the apoptotic mechanism in more detail, several survival factors were profiled. Induction of pro-apoptotic *APAF1*, *BAK*, *BIM*, and *BMF*, and loss of anti-apoptotic *BCLXL* occurred within 12 h of MSP treatment, in both HCT116 and HT29 colon cancer cell lines (Figure 2a). Induction of *BMF* was particularly striking, and was confirmed both at the mRNA and protein level in time-course studies (Figures 2b and c). Knockdown of Bmf using RNAi attenuated the increase in *BMF* mRNA levels following MSP treatment (Figure 2d), and there was a reduction in Bmf protein expression (Figure 2e). These changes were associated with reduced PARP cleavage and attenuated levels of cleaved caspases (Figure 2f). Notably, in cells treated with MSP, activated caspases 3, 6, and 9 were reduced to near background levels by Bmf knockdown. Cleaved caspase-8 also was detected after MSP treatment and this caspase apparently was unaffected by Bmf knockdown (Figure 2f). Further experiments should seek to optimize Bmf knockdown beyond the ~50–60% reduction achieved here (Figures 2d and e), and clarify the relative contributions of internal and external apoptotic pathways. Nonetheless, we conclude that Bmf was a key mediator of MSP-induced apoptosis in colon cancer cells.

### *BMF* activation by MSP involves HDAC8 and STAT3 de-recruitment

To prioritize histone marks for *BMF* chromatin immunoprecipitation (ChIP) assays, immunoblotting was first performed on whole-cell lysates of colon cancer cells. At the early time point of 3 h, MSP increased histone H3 acetylation (for example, H3acK9,14, H3acK9, and H3acK18) without dramatic changes in histone H3 methylation or phosphorylation (Supplementary Figure 1a). Histone H4 hyperacetylation also was detected within 3 h of MSP treatment, as evidenced by increased H4acK12 and H4acK5,8,12,16 histone marks (Supplementary Figure 1b). Time-course studies confirmed that histone hyperacetylation was maintained for several hours in response to MSP treatment, and that histone methylation marks associated with gene activation, such as H3K4me3, became prominent after 24 h (Supplementary Figure 1c). These findings indicated that MSP induced global histone acetylation before alterations in other histone marks.

**Figure 3:**
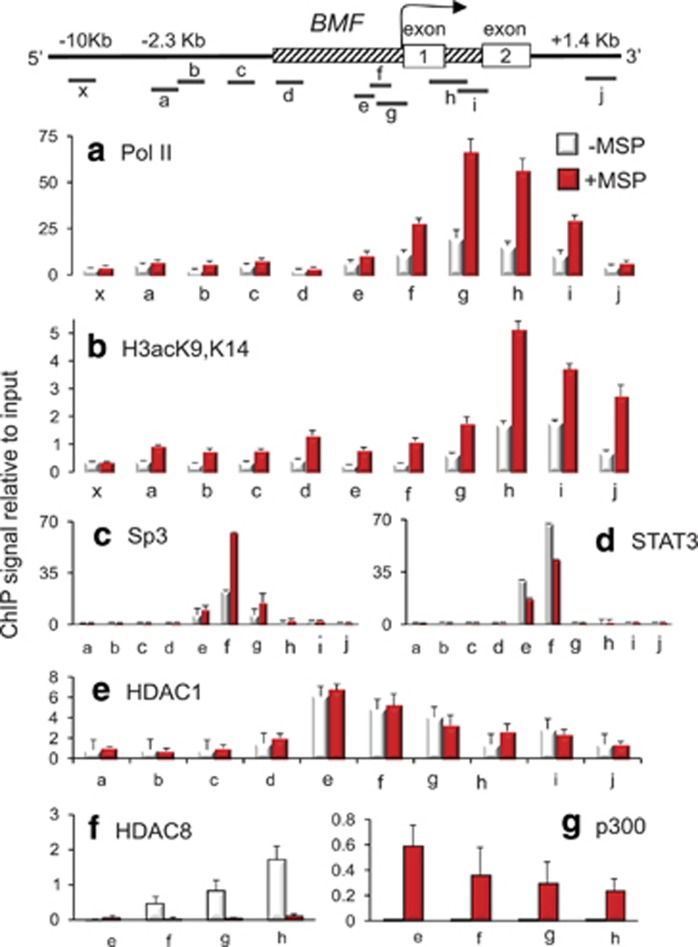
HDAC8 is a repressor of the *BMF* gene. Primers were designed to interrogate different regions of the *BMF* gene, including the promoter (hatched box) and flanking sequences. (**a**) MSP treatment enhanced RNA Pol II recruitment and (**b**) local histone acetylation levels. (**c**) MSP increased Sp3 transcription factor binding, adjacent to the transcription start site (arrow), and (**d**) reduced STAT3 associations. (**e**) HDAC1 interactions with the *BMF* promoter were unaffected by MSP treatment. (**f**) HDAC8 was present on *BMF* under constitutive conditions and was de-recruited following MSP treatment, coincident with increased p300 associations (**g**). ChIP assays were conducted at 4 h in HCT116 cells treated with MSP (+MSP) or vehicle (−MSP). Data were normalized to input controls; H3acK9,K14 also was normalized to histone H3. Results in **a**–**g** indicate mean±S.D., *n*=3, from a single experiment that was repeated on three or more separate occasions

Because global histone acetylation changes preceded alterations in other histone modifications, we conducted ChIP assays in colon cancer cells at an early time point, 4 h. Consistent with the *BMF* induction by MSP (Figure 2b), RNA polymerase II (Pol II) was recruited (Figure 3a) and there was a marked increase in local histone acetylation (Figure 3b). In parallel analyses of *BCLXL* (Supplementary Figure 2), reduced histone acetylation and attenuated Pol II levels were detected on the corresponding gene promoter after MSP treatment, consistent with the observed transcriptional downregulation of this pro-survival factor (Figure 2a). We did not examine serine 2-phosphorylated Pol II in association with *BCLXL* or *BMF*, but this might be included in future experiments.^20^

The *BMF* promoter has predicted binding sites for multiple transcription factors, including Sp1 (specificity protein 1)/Sp3 (specificity protein 3) and signal transducer and activator of transcription 3 (STAT3). ChIP assays with the corresponding antibodies failed to identify consistent associations for Sp1, but upstream of the transcriptional start site there was binding of Sp3 and STAT3 (region ‘f', Figures 3c and d). MSP treatment enhanced Sp3 and attenuated STAT3 interactions.

A prior report implicated HDAC1 as a repressor of *BMF* transcriptional activity.^9^ We confirmed HDAC1 interactions on the *BMF* promoter, slightly upstream of the transcription start site (peak region ‘e', Figure 3e). The interactions of HDAC1 with *BMF* did not appear to be altered by MSP treatment. We also detected HDAC8 associations under constitutive conditions in colon cancer cells (open bars, peak region ‘h', Figure 3f). To our knowledge, *BMF* is the first identified direct target gene for HDAC8 repression, none having been reported before.^17^ HDAC8 interactions were markedly reduced in response to MSP treatment (red bars, Figure 3f), and this coincided with increased histone acetyltransferase (HAT) p300 interactions (Figure 3g). Immunoblotting of whole-cell lysates showed no specific loss of HDAC8, or other class I HDACs, in response to MSP treatment (Supplementary Figure 3). We concluded that HDAC1 and HDAC8 might cooperate to maximally repress *BMF*, but HDAC8 de-recruitment alone was sufficient to activate the target gene.

### Inhibition of p300 or overexpression of HDAC8 interferes with *BMF* induction

**Figure 4:**
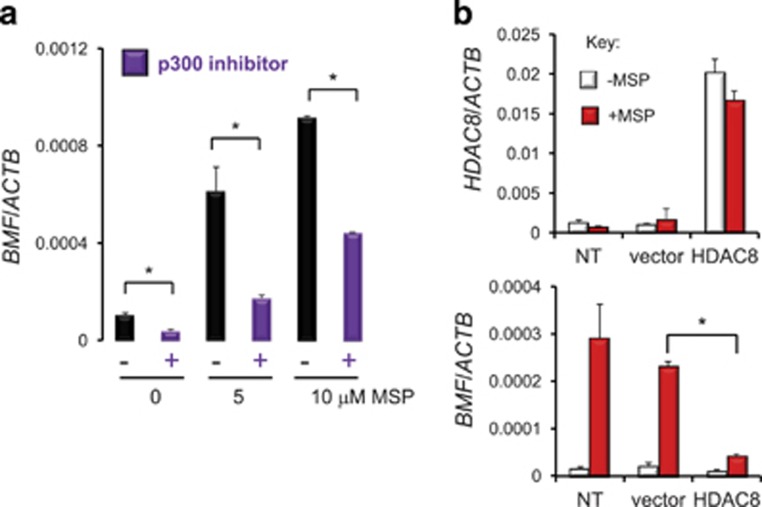
Inhibition of p300 and HDAC8 overexpression interferes with *BMF* transcription. (**a**) Pretreatment of colon cancer cells with p300 inhibitor C646^46^ (10 *μ*M) reduced the constitutive levels of *BMF* and interfered with induction of the target gene by MSP. (**b**) Forced expression of HDAC8 by transient transfection rescues cells from *BMF* induction by MSP. In **a**, **b**, bars indicate mean±S.D., *n*=3, from a single experiment that was repeated at least twice (*P<0.05, by Student's *t*-test)

On the basis of the ChIP data (Figures 3f and g), HDAC8 and p300 were examined further as regulators of *BMF* transcription. Treatment of colon cancer cells with a p300 inhibitor attenuated significantly the basal expression levels as well as the dose-dependent induction of *BMF* by MSP (Figure 4a). In transient transfection experiments, forced expression of HDAC8 also interfered with the ability of MSP to induce the target gene (Figure 4b), supporting a reciprocal relationship between p300 and HDAC8 in regulating *BMF* transcriptional activity. Although the results in Figure 4a were optimized for the specific p300 inhibitor selected, incomplete reductions in *BMF* expression were obtained, suggesting the possible involvement of other HATs in regulating *BMF*. Gain- and loss-of-function experiments, including the use of HAT mutants and HDAC8 silencing, might provide additional insights.

### HDAC8 and STAT3 cooperate to repress *BMF* transcription

**Figure 5:**
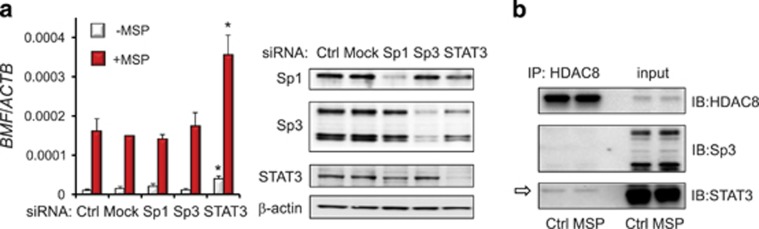
STAT3 associates with HDAC8 and represses *BMF* transcription. (**a**) siRNAs were used to target STAT3, Sp1, or Sp3, with transfection reagent (mock) and untreated controls (Ctrl) included in the experiment. After 48 h RNAi knockdown, cells were treated with MSP or vehicle for 12 h and *BMF* expression was determined relative to *ACTB*. Data=mean±S.D., *n*=3, from a single experiment that was repeated twice (*P<0.05, by Student's *t*-test). Knockdown of STAT3, Sp1, and Sp3 was confirmed by immunoblotting (IB). (**b**) Immunoprecipitation (IP) with HDAC8 antibody followed by IB with the corresponding antibodies identified a band for STAT3 (open arrow) but not Sp3 or Sp1 (data not shown). Results are representative of the findings from three independent experiments

To clarify the apparent reciprocal relationship between Sp3 and STAT3 in regulating *BMF* (Figures 3c and d), we performed RNAi experiments on the corresponding transcription factors. Knockdown of STAT3 increased the constitutive and MSP-induced levels of *BMF*, supporting a suppressive role for STAT3 on the *BMF* promoter (Figure 5a). Unexpectedly, knockdown of Sp3 did not produce a corresponding decrease in *BMF* expression, nor was there an apparent compensatory mechanism involving Sp1 upregulation after Sp3 silencing (Figure 5a). These results suggested a possible ‘bystander' effect in which Sp3 interacts with a cognate binding site that becomes unmasked following STAT3 de-recruitment, without necessarily activating the target gene. We conclude that in addition to STAT3 and Sp3, other factors regulate *BMF* transcriptional activity on HDAC8 release, possibly in association with HDAC1.

**Figure 6:**
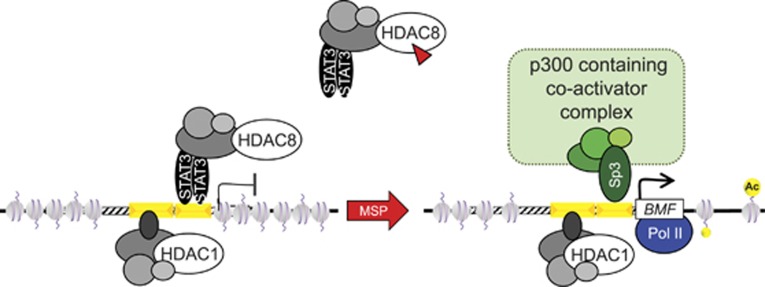
Model for HDAC regulation of *BMF* transcriptional activity. MSP inhibits HDAC8 more effectively than HDAC1,^10^ which triggers the release of a corepressor complex comprising HDAC8, STAT3, and other partners. Release of the HDAC8 corepressor complex recruits p300, Sp3, and other factors that drive RNA Pol II activity

Finally, HDAC8 was immunoprecipitated from whole-cell lysates of HCT116 cells and immunoblotted for potential protein partners. No interaction with Sp3 was detected, but a band corresponding to STAT3 was identified (Figure 5b, open arrow). To our knowledge, this is the first evidence reported for a protein partner in the HDAC8 corepressor complex.^17^ Notably, the STAT3 band was detected in the presence and absence of MSP treatment (Figure 5b), suggesting that an HDAC8/STAT3 corepressor complex might be released from the *BMF* promoter to recruit p300 and other coactivators (Figure 6).

## Discussion

The first X-ray crystallography structures published of a human HDAC, HDAC8,^13,14^ provided valuable insights into the catalytic mechanism and binding characteristics of various inhibitors. A decade later, surprisingly little information was available on the role of HDAC8 in physiology or pathophysiology. HDAC8 associates with *α*-actin and regulates smooth muscle contractility,^21^ as well as influencing human myometrial activity via the acetylation of heat–shock protein 20.^22^ Other class I HDACs (HDAC1, HDAC2, and HDAC3) are known to associate with gene promoters in repressor complexes, but promoter interactions with HDAC8 have not been reported, nor have regulatory gene targets of HDAC8 been clearly described.^17^ We now report, for the first time, that HDAC8 can associate with the *BMF* gene under constitutive conditions in colon cancer cells, and establish a pro-survival scenario via the repression of Bmf protein levels.

The increase in global histone acetylation in colon cancer cells treated with MSP was recapitulated in several other cancer cell lines. For example, time-course studies in BE(2)C neuroblastoma cells revealed a rapid induction of histone acetylation by MSP compared with MSC parent compound (Supplementary Figure 4a). Interestingly, MSC, MSP, and the structural analog *β*-keto-methylselenobutyrate (KMSB) also induced global histone acetylation in prostate, breast, lung, and leukemia cells, whereas selenomethionine (SM) had no such effect (Supplementary Figure 4b). Unlike MSC, SM is a poor substrate for aminotransferase activity.^12^ In a pilot study *in vivo*, we observed that dietary MSC, but not SM, inhibited mouse colon tumor multiplicity, and this was associated with increased Bmf expression and histone acetylation in the tumors, without marked changes in overall HDAC8 protein levels (Supplementary Figure 5). We are now seeking to test the *in vivo* efficacy of MSP and KMSB along with the corresponding parent compounds as inducers of Bmf. Further insights also might come from ChIP assays following treatment with MSC and a transaminase inhibitor, such as aminooxyacetic acid (AOAA; Figure 1); however, such experiments would have to be optimized to account for the time taken to convert MSC to MSP at levels that might inhibit HDAC activity.^10,11^ We did not specifically examine changes in *BMF* expression or HDAC promoter occupancy in other cell lines shown (Supplementary Figure 4), or in colon cancer cells other than HCT116 and HT29 (Figure 2a). Such experiments might help to clarify the working model proposed here.

A notable feature of the working model for MSP (Figure 6) is the continued interaction of HDAC1 on the *BMF* promoter following the release of HDAC8. Time-course ChIP assays might indicate whether HDAC1 is released at later times, perhaps coinciding with the appearance of activating histone methylation marks, such as H3K4me3 (Supplementary Figure 1c). It would be interesting to assess whether, over time, other HDACs substitute at the position normally occupied by the HDAC8 complex to downregulate the target gene. This could have implications for therapeutic strategies selectively targeting HDAC8 *versus* other HDACs. HDAC8 alone was implicated in the pathogenesis of neuroblastoma,^19^ but multiple HDACs are dysregulated in most other cancers.^17,18^ ChIP/re-ChIP assays in the presence of agents such as etinostat, which preferentially target class I HDACs other than HDAC8,^17^ might provide insights into the roles of HDAC1, HDAC2, and HDAC3 in regulating *BMF* expression in the absence of HDAC8. Studies with other HDAC inhibitors also might identify changes in the composition of the corepressor complex that mask specific HDAC associations, rather than necessarily triggering HDAC release from the *BMF* gene promoter.

In the case of vorinostat (suberoylanilide hydroxamic acid, SAHA), *BMF* was highly induced compared with MSP under the same conditions at 4 h, and there was de-recruitment of both HDAC1 and HDAC8 (Supplementary Figure 6). SAHA is an established ‘pan-HDAC' inhibitor,^17,18^ whereas MSP has 10-fold greater inhibitory activity toward HDAC8 than HDAC1.^10^ Molecular modeling revealed that residue W141 in HDAC8 is replaced by L141 in HDAC1, and this results in less favorable docking of MSP in the active site (Supplementary Figure 7). We speculate that structural features allow MSP to preferentially target HDAC8 for release according to the working model (Figure 6), whereas pan-HDAC inhibitors like SAHA de-recruit both HDAC1 and HDAC8 corepressor complexes to maximally induce the *BMF* target gene (Supplementary Figure 8).

ChIP assays also revealed that SAHA had little effect on Sp3 interactions, whereas STAT3 levels were markedly reduced on the *BMF* promoter (Supplementary Figures 6c and d, black *versus* white bars, region ‘f'). These findings further support the ‘bystander' role of Sp3, proposed above, and the repressive actions of STAT3 on the target gene. Co-IP experiments identified a modest increase in STAT3 acetylation following MSP and SAHA treatment (Supplementary Figure 9a), suggesting a possible role for post-translational modifications to STAT3. However, in HCT116 cells expressing knock-in mutants of STAT3, a STAT3 phosphorylation mutant that interferes with DNA interactions^23^ consistently enhanced the basal levels of *BMF*, whereas a STAT3 acetylation mutant^24^ had less impact on the target gene (Supplementary Figure 9b). Interestingly, in all three cell lines, there was a comparable time-dependent increase in *BMF* expression that plateaued between 6 and 24 h after MSP treatment (Supplementary Figure 9b). We interpret these data as evidence that, beyond STAT3 post-translational modifications, HDAC8 inhibition and de-recruitment likely serves as the primary driver of *BMF* transcriptional activity in response to MSP.

Prior work established that the silencing of *HDAC8* by RNAi activated apoptosis and/or inhibited the growth of human cancer cells.^25,26^ No significant changes in *BMF* mRNA levels were detected after treatment of colon cancer cells with 5-aza-2'-deoxycytidine (Supplementary Figure 10), implying that DNA promoter methylation did not have a critical role in silencing *BMF* transcription under the conditions reported here.

Finally, the current findings provide possible new leads for understanding the biological roles of HDAC8 in schistosomiasis,^27^ chronic obstructive pulmonary disease,^28,29^ and skull morphogenesis.^30,31^ Interestingly, novel mutations affecting *HDAC8* and *EP300* recently were linked to the development of Cornelia de Lange syndrome (CdLS), Rubinstein–Taybi syndrome, and other congenital abnormalities.^32, 33, 34, 35^ Loss-of-function *HDAC8* mutations in six CdLS probands increased the acetylation and loading onto chromatin of SMC3, a ‘cohesin' that has a role in regulating gene expression.^32^ Another member of the cohesin complex, *NIPBL* (nipped-B-like), is commonly mutated in patients with CdLS, and the corresponding loss-of-function zebrafish embryos have increased apoptosis in developing neural tissues.^34^ Chemical activation of the pathway in *nipblb*-loss-of-function embryos rescued the adverse phenotypes and restored the physiological levels of cell death.^34^ This is consistent with the working model proposed here (Figure 6), in which functional HDAC8 associates with, and represses, pro-apoptotic *BMF*, whereas loss-of-function HDAC8 mutants would aberrantly activate apoptosis and have deleterious consequences during embryo development. On the basis of the results presented here, we speculate that dysregulation of Bmf-mediated apoptosis might have a critical role in CdLS and related syndromes.

## Materials and Methods

### Cell culture

HCT116^p21+/+^ and HCT116^p21−/−^ cells were provided by Dr. Bert Vogelstein (Johns Hopkins University, Baltimore, MD, USA). Dr. Zhenghe Wang (Case Western Reserve University, Cleveland, OH, USA) kindly supplied HCT116 cells expressing acetylation and phosphorylation mutants of STAT3.^23,24^ All other cell lines were obtained from ATCC (Manassas, VA, USA) and were cultured under the conditions described elsewhere.^36, 37, 38, 39, 40, 41, 42, 43^ Chemicals were from the sources reported^10,11,43^ and were added to cells at the following nominal concentrations, unless stated otherwise: MSP (10 *μ*M), MSC (50 *μ*M), KMSB (10 *μ*M), SM (50 *μ*M), and SAHA (3 *μ*M). In some experiments, 0, 0.1, 0.5, or 2 mM concentrations of AOAA (Sigma, St. Louis, MO, USA) were added 1 h before other test compounds to inhibit the transamination reaction.^44,45^ Other experiments used a p300 inhibitor^46^ (10 *μ*M C646, Sigma), added to cells 30 min before MSP treatment, or DNA methyltransferase inhibitor 5-aza-2'-deoxycytidine, as reported.^40^ Cells were collected at the times indicated in the figures.

### Cell viability and cell cycle analyses

MTT assays and fluorescence-activated cell sorting analyses were conducted according to the basic protocols reported elsewhere.^47,48^

### Western blot

Whole-cell lysates were immunoblotted using the methodology described elsewhere.^36,37^ Primary antibodies were to the following acetylated (ac), methylated (me), and phosphorylated (ph) histone marks: H3K9me3, H3K4me2, H4acK12, and H4acK5,K8,K12,K16 (Upstate, Temecula, CA, USA); H3acK9,K14, H3acK9, and H3S10ph (Cell Signaling, Beverly, MA, USA); H3K27me3, H3K4me3, H3S28ph, and H3T3ph (Active Motif, Carlsbad, CA, USA); and H3acK18 (Abcam, San Francisco, CA, USA). Other primary antibodies were to histone H3 and histone H4 (Upstate, Cell Signaling, Active Motif); PARP, p21, Bmf, cleaved caspase-3, cleaved caspase-6, cleaved caspase-8 and cleaved caspase-9 (Cell Signaling); HDAC8 (R&D Systems, Minneapolis, MN, USA); HDAC1 and p300 (Santa Cruz Biotechnology, Dallas, TX, USA); and *β*-actin (Sigma). Antibodies to p53, acetylated p53, *α*-tubulin, acetylated *α*-tubulin, STAT3, Sp1, and Sp3 were as reported.^10,11,36,37,48, 49, 50, 51^

### Knockdown and overexpression experiments

HDAC8 overexpression was achieved by transient transfection of 0.5 *μ*g *HDAC8* construct or empty vector (OriGene, Rockville, MD, USA), using the protocol reported before for *HDAC3* and *HDAC6.*^36^ In knockdown studies, cells were transfected with Bmf siRNA (SASI_Hs01_00127817, Sigma) or negative control siRNA (Universal Negative control #1, Sigma) using Lipofectamine RNAiMAX (Invitrogen, Carlsbad, CA, USA), according to the manufacturer's instructions. After 48 h, cells were treated with MSP or vehicle and collected at 12 h. RNAi also was used as reported^48, 49, 50, 51^ to knockdown Sp1, Sp3, and STAT3.

### Real-time RT-PCR and qPCR

Methodologies for RNA extraction, cDNA synthesis, and qPCR were reported before.^10,40,41^ PCR reactions were performed on a Roche Light Cycle 480 II instrument (Roche, Indianapolis, IN, USA). Primers were to the following targets: *BMF*, *BCL2*, *BCLXL*, *BAX*, *BAK*, *BIM*, *APAF1*, and *P21WAF1*. Targets were normalized to internal controls indicated in the figures.

### Chromatin immunoprecipitation

After treatment with MSP or vehicle for 4 h, cells were incubated with 1% formaldehyde in cell culture medium for 10 min at room temperature, and then the reaction was stopped with 0.12 M glycine for 5 min. DNA shearing and ChIP used the ChIP-IT Express Enzymatic (Active Motif) kit, following the recommended protocol. In the IP step, anti-histone H3, anti-H3acK9,K14 (Cell Signaling), and anti-RNA polymerase II (Active Motif) were incubated overnight at 4 °C. Additional IP experiments used antibodies to Sp1, Sp3, STAT3, HDAC8, HDAC1, and p300 (from sources listed above). After eluting pull-down DNA from magnetic beads, DNA purification used the QIAquick PCR Purification kit (Qiagen, Valencia, CA, USA). Quantification of immunoprecipitated and input (control) DNA was carried out by qPCR on a Light Cycler 480 II (Roche), with Light Cycle 480 SYBR Green I Kit master (Roche). The primer sequences are available on request.

### Molecular modeling, docking, and pocket binding prediction

The 3D coordinates of the human catalytic domain of HDAC8 in the inhibited conformation and HDAC1 in the active conformation were retrieved from the Protein Data Bank (PDB 1T67 and 4BKX, respectively). The human catalytic domain of HDAC1 in the inhibited conformation was built through homology modeling using PDB 1767 as a starting template (Molsoft ICM 3.7-2 d, Molsoft, San Diego, CA, USA).^52^ Models were energetically refined in the internal coordinate space with Molsoft ICM.^36,53^ Five types of interaction potential were considered: (i) van der Waals potential for a hydrogen atom probe; (ii) van der Waals potential for a heavy-atom probe (generic carbon of 1.7 Å radius); (iii) optimized electrostatic term; (iv) hydrophobic terms; and (v) loan pair-based potential, which reflects directional preferences in hydrogen bonding. Energy terms were based on the Merck molecular force field to account for solvation free energy and entropic contribution. Modified intermolecular terms, such as soft van der Waals, hydrogen bonding, and hydrophobic terms, were included in the analyses. Conformational sampling is based on the biased probability Monte Carlo procedure, which randomly selects a conformation in the internal coordinate space and then moves randomly to a new position independent of the previous one, according to a predefined continuous probability distribution. After each random step, full local minimization improves the efficiency of the procedure. In the ICM-VLS (Molsoft ICM) screening procedure, ligand scoring is optimized to obtain maximal separation between binders and non-binders. Ligand was assigned a score according to its fit within the protein, accounting for electrostatic, hydrophobicity, and entropy parameters.^36,53^ The protocol was first validated by docking trichostatin A into the binding site and reproducing the crystallographic pose in HDAC8 (PDB 1T64). Binding-pocket volumes were predicted using Pocket Finder (Molsoft ICM 3.7-d).^54,55^

### Statistical analyses

Unless indicated otherwise data were expressed as mean±S.D. (*n*=3). Paired comparisons were made using Student's *t*-test. A *P*-value <0.05 was considered as statistically significant, and indicated as such with an asterisk in the corresponding figures.

## Abbreviations

Bmf/*BMF*: Bcl-2-modifying factor protein/gene
CdLS: Cornelia de Lange syndrome
ChIP: chromatin immunoprecipitation
HAT: histone acetyltransferase
HDAC: histone deacetylase
KMSB: *β*-keto-methylselenobutyrate
MSC: methylselenocysteine
MSP: methylselenopyruvate
MTT: 3-(4,5-dimethylthiazol-2-yl)-2,5-diphenyltetrazolium bromide
*NIPBL*: nipped-B-like
STAT3: signal transducer and activator of transcription 3
PARP: poly(ADP-ribose)polymerase
RNA Pol II: RNA polymerase II
SAHA: suberoylanilide hydroxamic acid
SM: selenomethionine
Sp1: specificity protein 1
Sp3: specificity protein 3
