# Supplementary material for: HDAC8 and STAT3 repress BMF gene activity in colon cancer cells
Source: Cell Death Dis. 2014 Oct 16;5(10):e1476–. doi: 10.1038/cddis.2014.422 (PMC4237248; doi:10.1038/cddis.2014.422)
Supplement: Supplementary Figure Legends [file cddis2014422x1.doc]

### Supplementary Results

**HDAC8 and STAT3 repress *BMF* gene activity in colon cancer cells**

Y Kang, H Nian, P Rajendran, E Kim, WM Dashwood, JT Pinto, LA Boardman, SN Thibodeau, PJ Limburg, CV Löhr, WH Bisson, DE Williams, E Ho, RH Dashwood

**Supplementary Figure 1** MSP-induced histone acetylation precedes changes in other histone marks. (**a**) MSP increased global histone H3 acetylation at 3 h, without markedly altering global histone methylation or phosphorylation. (**b**) MSP also induced histone H4 acetylation within 3 h. (**c**) Other gene activation marks were induced by MSP, at later times, such as dimethylation and trimethylation of histone H3K4 (H3K4me2, H3K4me3). Histones H3 and H4 served as loading controls for the corresponding immunoblots.

**Supplementary Figure 2** MSP attenuates histone acetylation on the promoter region of a pro-survival gene, *BCLXL*. Primers were designed to interrogate the *BCLXL* promoter immediately upstream of the transcription start site. In HCT116 cells, MSP lowered RNA Pol II levels and histone H3K9,K14 acetylation status, consistent with the reduced gene expression data shown in Fig. 2a. Results indicate mean±SD, n=3, from a single ChIP assay that was repeated twice; **P*<0.05, by Student’s *t*-test.

**Supplementary Figure 3**  MSP treatment does not trigger early turnover of HDACs. Whole cell lysates of HCT116 and HT29 cells were immunoblotted 8 h after treatment with MSP or vehicle. Results are representative data from two or more experiments.

**Supplementary Figure 4** Seleno-α-keto acids induce histone acetylation in multiple cancer cell lines. (**a**) In neuroblastoma cells, MSP induced histone acetylation within 2 h, compared with >6 h for the parent compound MSC. (**b**) MSC, MSP, and the structural analog KMSB induced global histone acetylation in various cancer cell lines. In contrast to MSC, SM is a poor substrate for the transaminase reaction and fails to induce histone acetylation in the cell lines indicated.

**Supplementary Fig 5.**  MSC inhibits colon carcinogenesis and increases histone acetylation and Bmf levels in tumors. (**a**) Male ICR mice (n=10/group) were treated with 20/mg kg 1,2-dimethylhydrazine (DMH) by *i.p.* injection, once a week for 10 weeks, as reported.^1^ MSC and SM were incorporated into AIN93G diet at 6 ppm^2^ and fed for 17 weeks post-DMH treatment. Tumor multiplicity data = mean±SD; **P*<0.05 by Student’s *t*-test. (**b**) Colon tumors (T) and adjacent normal looking colonic mucosa (N) from DMH-treated mice given dietary MSC, as well as control colon tissues (Ctrl) from untreated animals, were prepared as whole cell lysates and immunoblotted for the proteins shown. Experiments were approved by the Institutional Animal Care & Use Committee.

**Supplementary Figure 6** *BMF* induction by a pan-HDAC inhibitor is associated with HDAC1, HDAC8, and STAT3 de-recruitment. Primers for ChIP assays were used to interrogate (**a**) RNA Pol II, (**b**) H3acK9,K14, (**c**) Sp3, (**d**) STAT3, (**e**) HDAC1 and (**f**) HDAC8 associations with different regions of the *BMF* gene, as detailed in Fig. 3. (**g**) qRT-PCR assays were used to determine *BMF* mRNA levels relative to *ACTB*, following MSP and SAHA treatment. Data = mean±SD, n=3, from experiments repeated three or more times. Open arrow, time-point at which ChIP assays in (a)-(f) were performed.

**Supplementary Figure 7** *In silico* modeling supported enhanced binding of MSP with HDAC8 *versus* HDAC1. (**a**) MSP (carbon atoms in purple) docked into the human HDAC8 active site binding pocket (PDB:1T67). Protein residues are displayed as sticks with carbon atoms in green; zinc, cyan-colored sphere. (**b**) HDAC8 residue W141 (colored green) is replaced by HDAC1 residue L141 (colored red), which increases the binding pocket volume (red shaded area) and does not allow for optimal orientation of the α-keto acid moiety in MSP (carbon atoms in black) to interact with the zinc (cyan-colored sphere). Also displayed is the orientation of residue L139 (orange) in the active conformation of HDAC1 (PDB:4BKX). For docking parameters and pocket binding predictions, see text and Nian et al.^3^

**Supplementary Figure 8** Model for pan-HDAC inhibitor regulation of *BMF* transcription. In contrast to MSP, which preferentially targets HDAC8 for early release (Fig. 6), SAHA and other pan-HDAC inhibitors also de-recruit the HDAC1 corepressor complex, resulting in greater activation of the target gene. TF, additional transcription factor(s).

**Supplementary Fig 9** MSP induces STAT3 acetylation, but this does not critically influence *BMF* expression. (**a**) HCT116 cells were treated with MSP, SAHA, or vehicle (control, Ctrl), and 6 h later whole cell lysates were subjected to co-IP with antibodies shown in the figure. (**b**) HCT11 cells containing wild-type STAT3 or knock-in mutants of STAT3 that interfere with phosphorylation or acetylation^4,5^ were treated with MSP (10 μM), and at the times shown *BMF* expression was determined relative to *ACTB*.

**Supplementary Figure 10** A DNA methyltransferase inhibitor has no effect on *BMF* transcription. HCT116 cells were treated with 5-aza-2’-deoxycytidine (5Aza), as reported.^6^ At the times indicated, *BMF* expression was determined relative to *ACTB*. MSP was included as a positive control for induction of the target gene. Data = mean±SD, n=3, from a single representative experiment that was repeated twice.

**References**

### Sumiyoshi H, Wargovich MJ. Chemoprevention of 1,2-dimethylhydrazine-induced colon cancer in mice by naturally occurring organosulfur compounds. *Cancer Res* 1990; 50: 5085-5087.

### Kim A *et al*. Long exposure of non-cytotoxic concentrations of methylselenol suppresses the invasive potential of B16F10 melanoma. *Oncol Rep* 2008; 20: 557-565.

### Nian H, Bisson WH, Dashwood WM, Pinto JT, Dashwood RH. α-Keto acid metabolites of organoselenium compounds inhibit histone deacetylase activity in human colon cancer cells. *Carcinogenesis* 2009; 30: 1416-1432 .

### Zhang P *et al*. Cross-talk between phospho-STAT3 and PLCγ plays a critical role in colorectal tumorigenesis. *Mol Cancer Res* 2011; 9: 1418-1428.

### Lee P *et al*. Acetylated STAT3 is crucial for methylation of tumor-suppressor gene promoters and inhibition by resveratrol results in demethylation. *Proc Natl Acad Sci USA* 2012; 109: 7765-7769.

### Wang R *et al*. Epigenetic inactivation of endothelin-2 and endothelin-3 in colon cancer. *Int J Cancer* 2013; 132: 1004-1012.
