# Supplementary figures and images for: HDAC8 and STAT3 repress BMF gene activity in colon cancer cells
Source: Cell Death Dis. 2014 Oct 16;5(10):e1476–. doi: 10.1038/cddis.2014.422 (PMC4237248; doi:10.1038/cddis.2014.422)

## MSP induces histone acetylation prior to changes in other histone marks

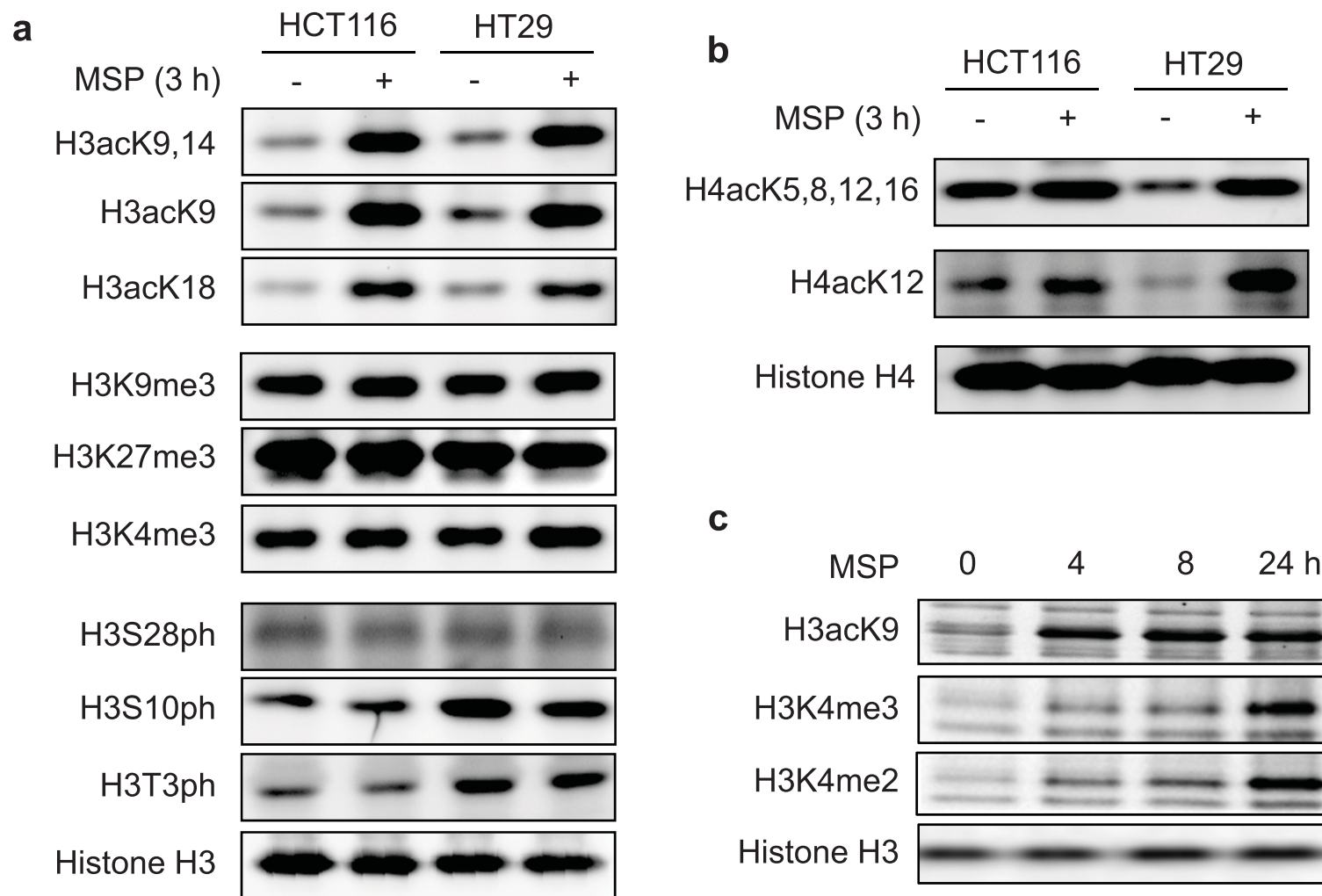

Supplementary Fig 1

Supplement: Supplementary Figure 1 [file cddis2014422x2.pdf]

MSP reduces histone acetylation and RNA pol II on the *BCLXL* promoter

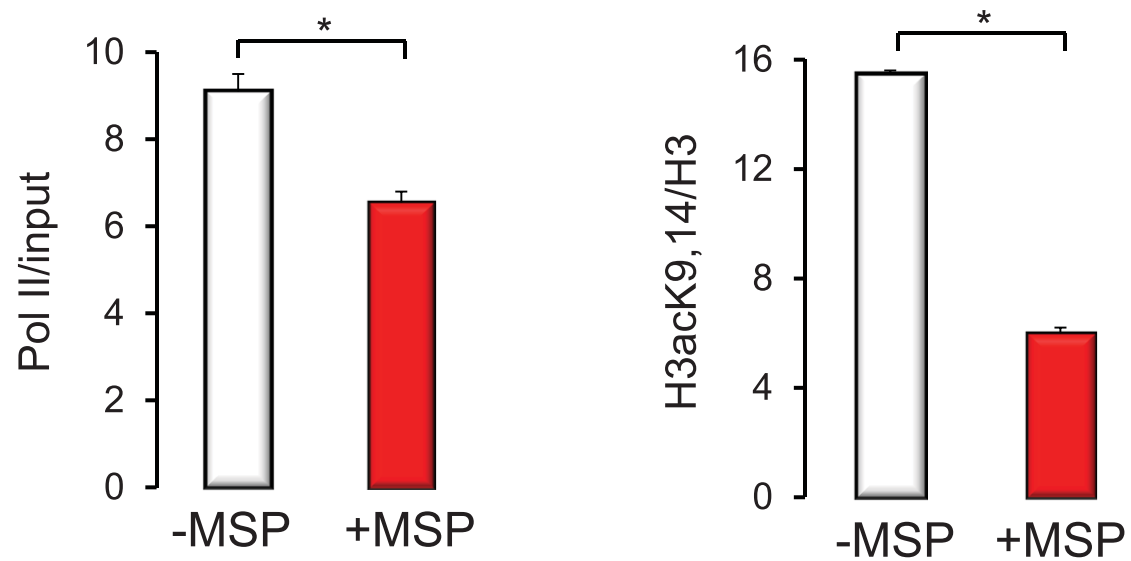

Supplement: Supplementary Figure 2 [file cddis2014422x3.pdf]

## MSP does not trigger class I HDAC turnover after 8 h

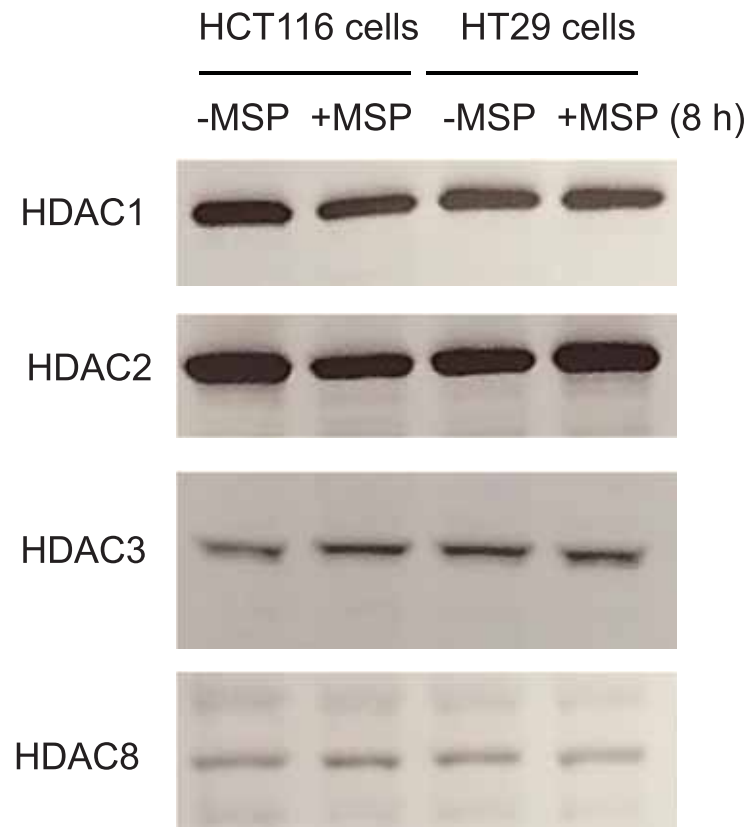

Supplement: Supplementary Figure 3 [file cddis2014422x4.pdf]

## MSP induces histone acetylation in multiple cancer cell lines

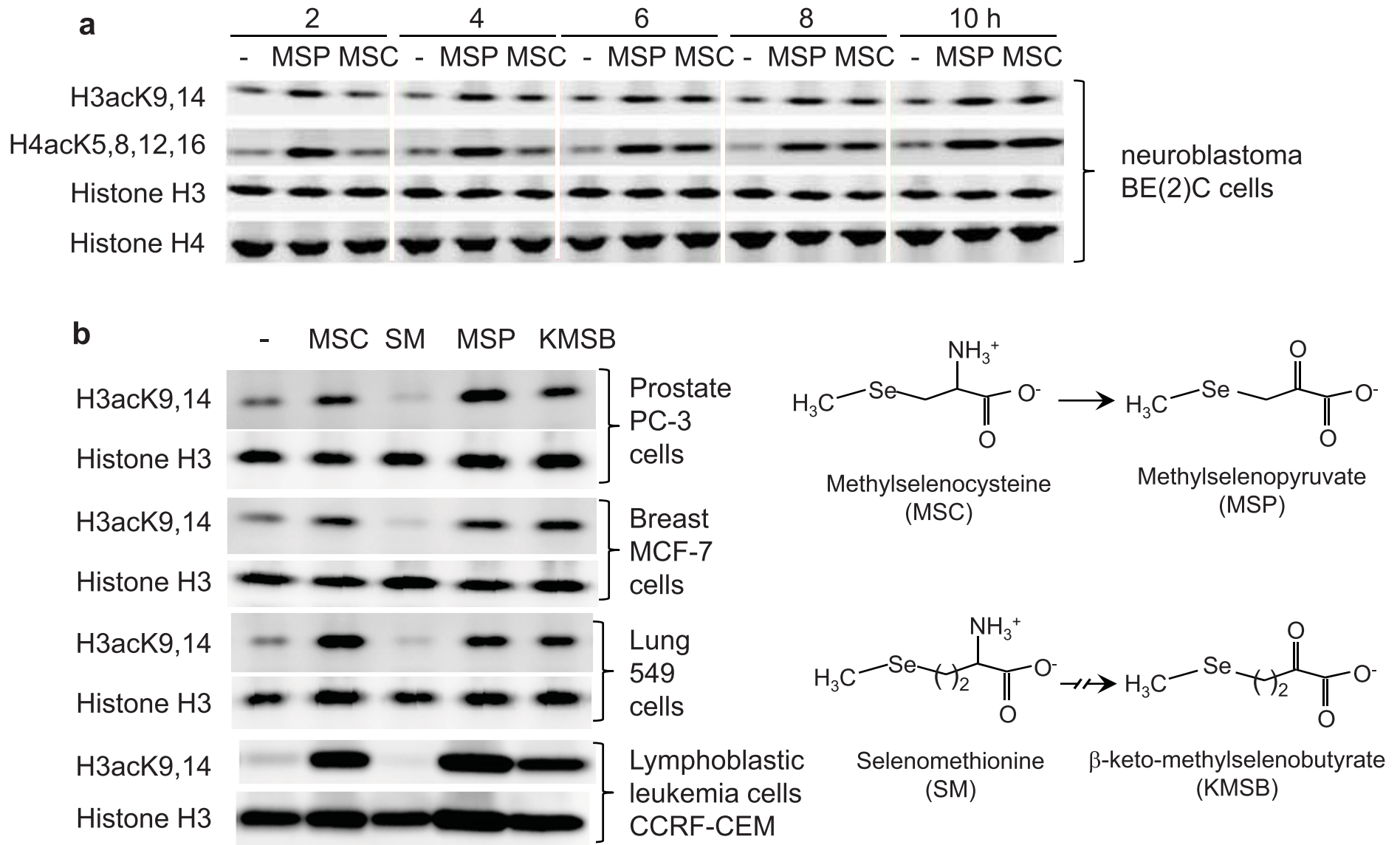

Supplementary Fig 4

Supplement: Supplementary Figure 4 [file cddis2014422x5.pdf]

## MSC inhibits colon carcinogenesis and induces Bmf in tumors

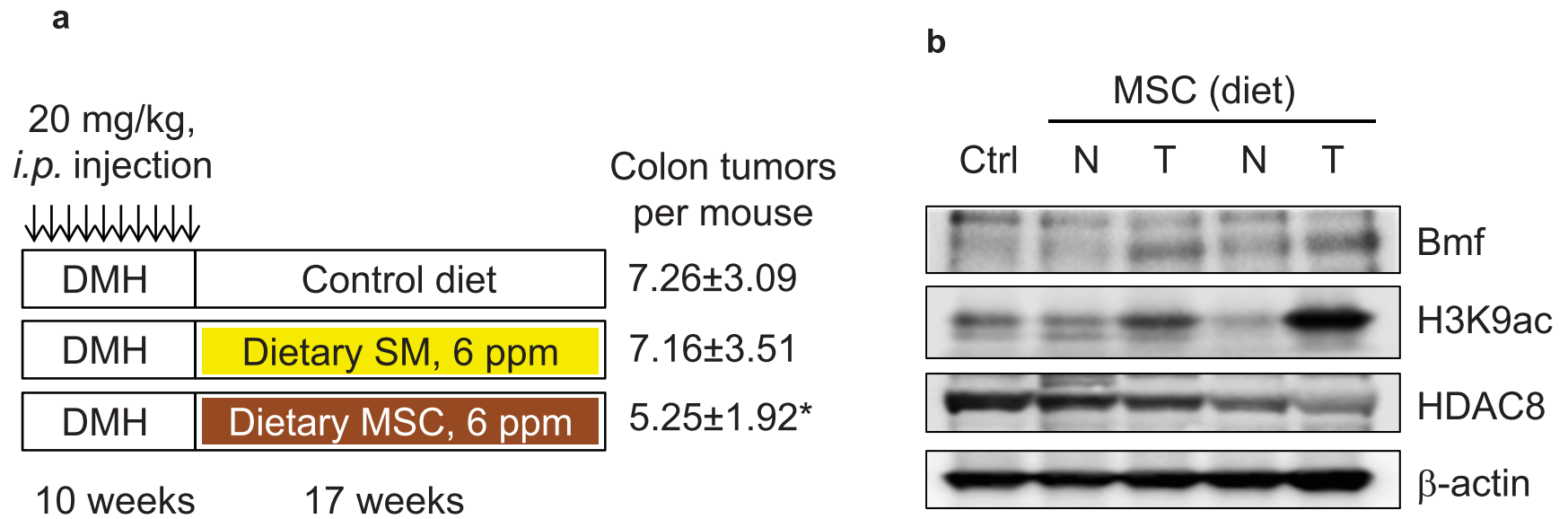

Supplement: Supplementary Figure 5 [file cddis2014422x6.pdf]

# BMF induction by SAHA involves loss of HDAC1, HDAC8, and STAT3

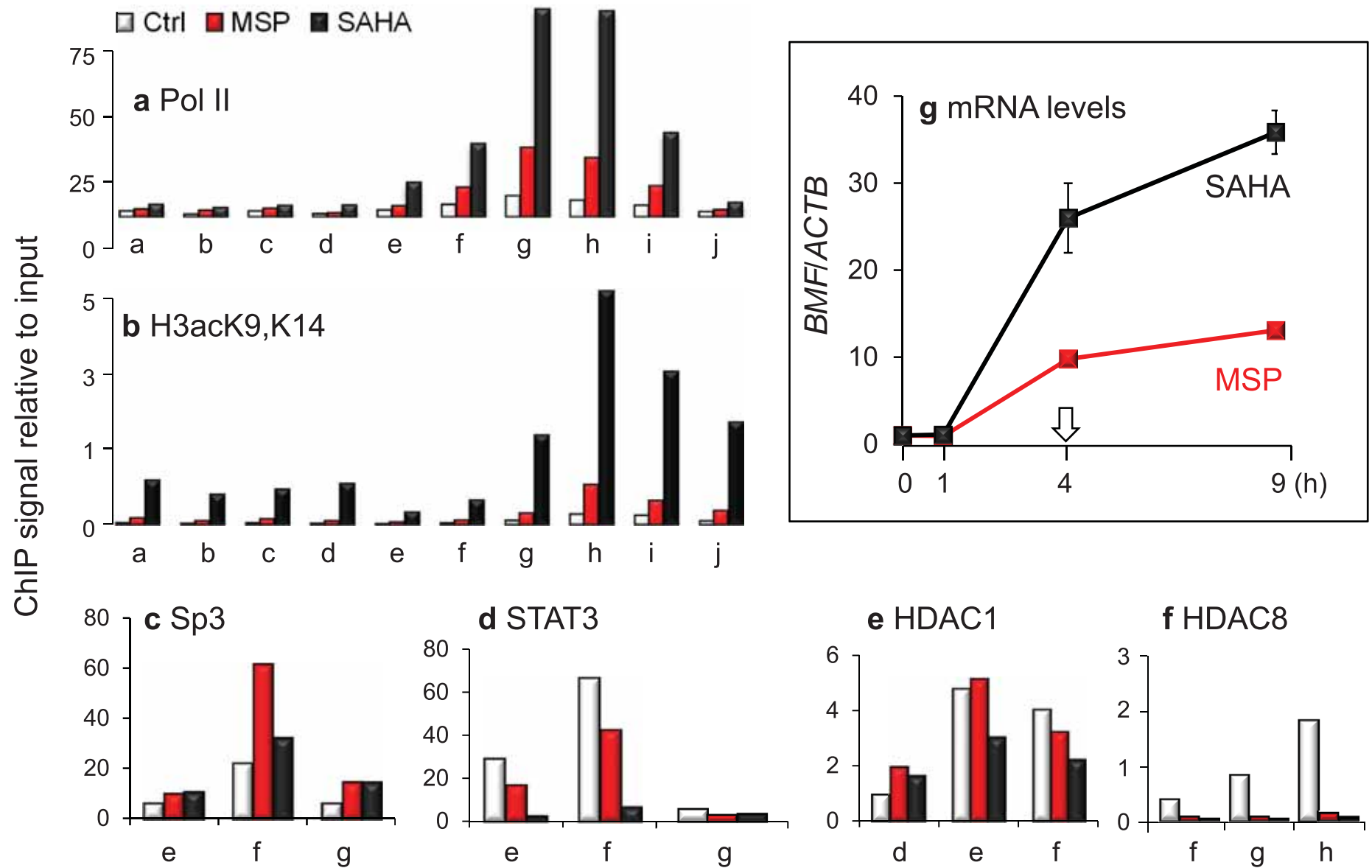

Supplementary Fig 6

Supplement: Supplementary Figure 6 [file cddis2014422x7.pdf]

## MSP interacts more favorably with HDAC8 than HDAC1

**a**

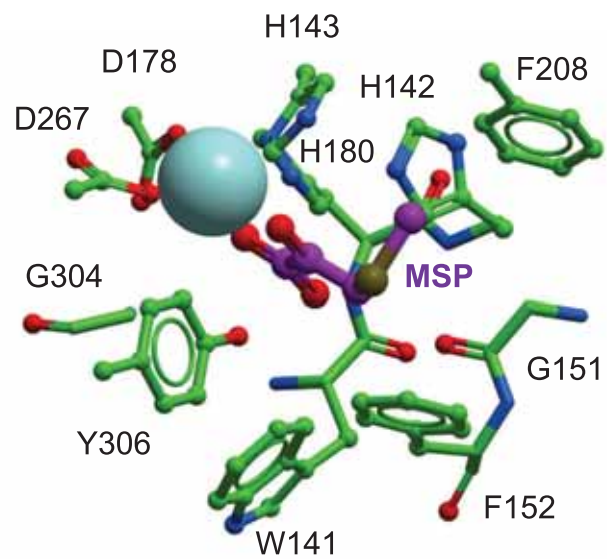

**b**

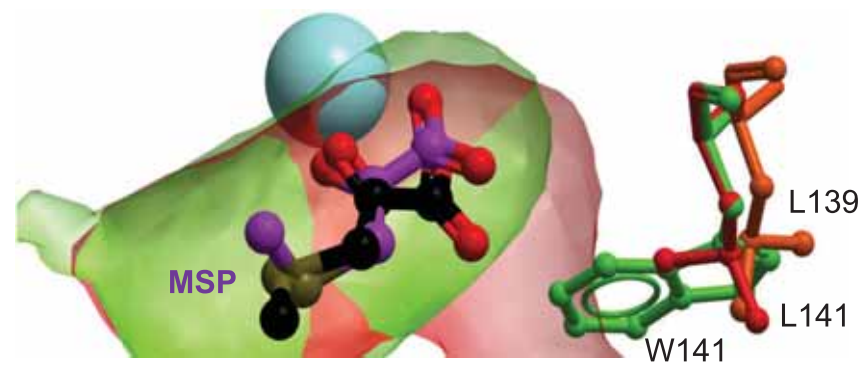

Supplement: Supplementary Figure 7 [file cddis2014422x8.pdf]

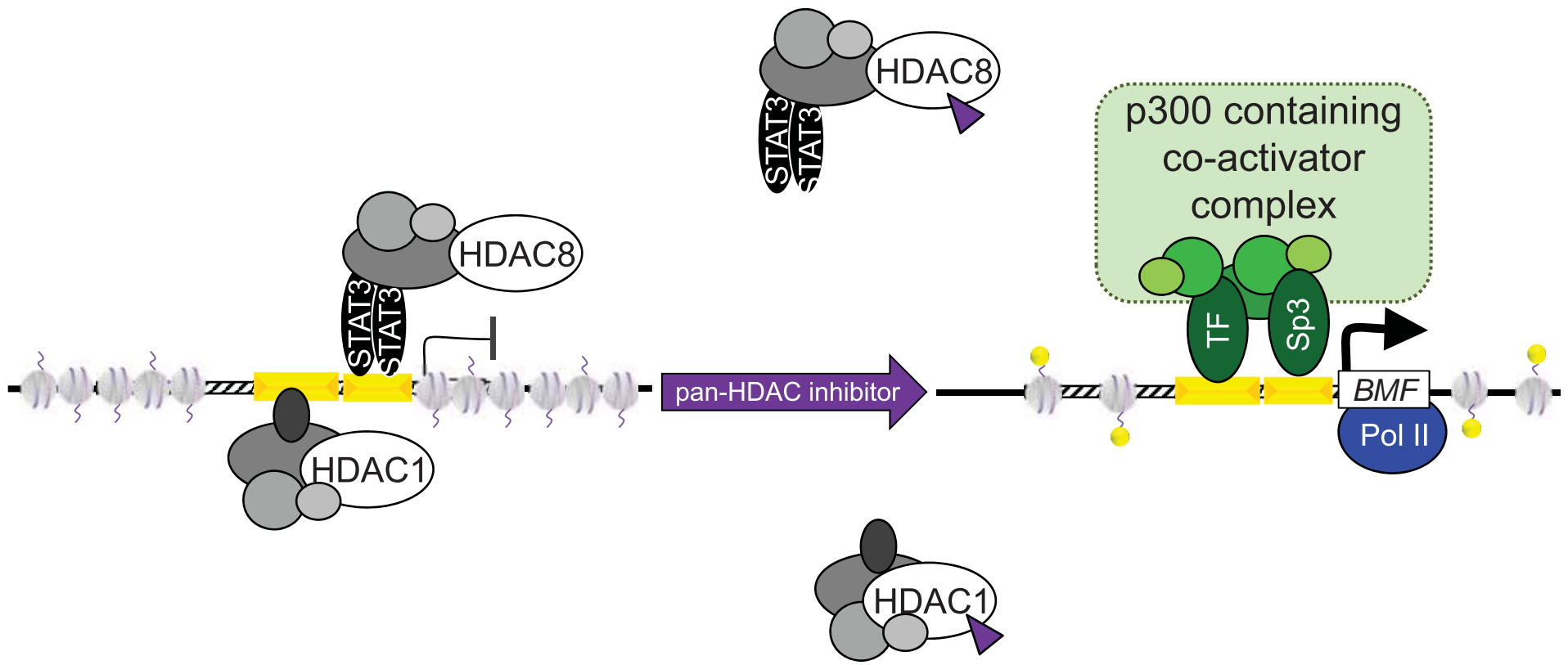

Supplementary Fig 8

Supplement: Supplementary Figure 8 [file cddis2014422x9.pdf]

## No role for DNA methylation in silencing *BMF*

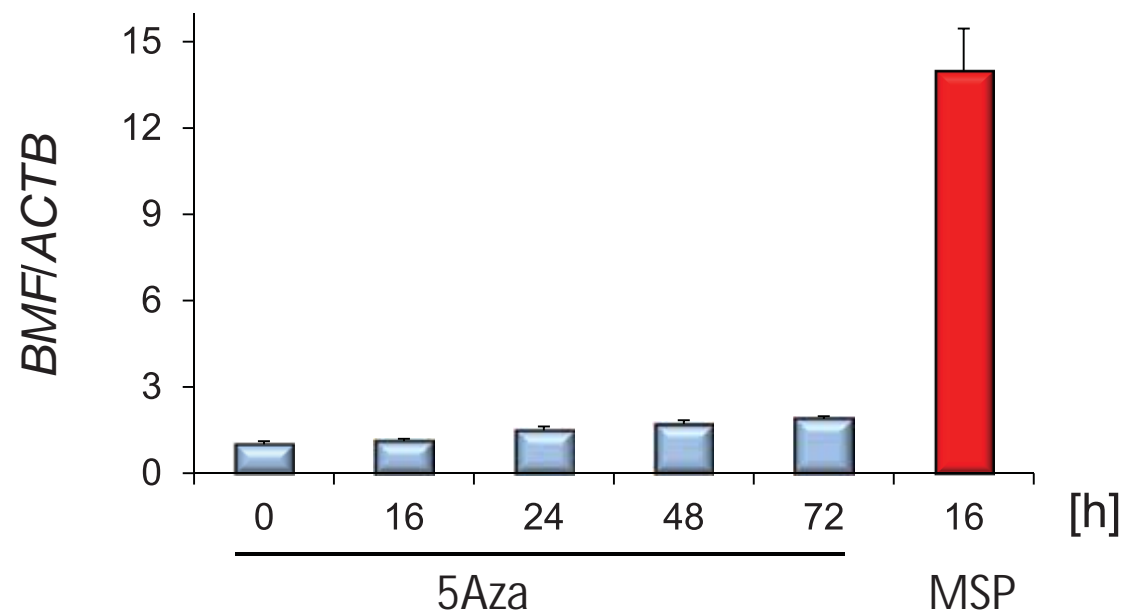

Supplement: Supplementary Figure 10 [file cddis2014422x11.pdf]
