# Supplementary material for: HDAC8 and STAT3 repress BMF gene activity in colon cancer cells
Source: Cell Death Dis. 2014 Oct 16;5(10):e1476–. doi: 10.1038/cddis.2014.422 (PMC4237248; doi:10.1038/cddis.2014.422)
Supplement: Supplementary Figure 9 [file cddis2014422x10.pdf]

STAT3 acetylation is not a key determinant of MSP-induced *BMF* activation

a

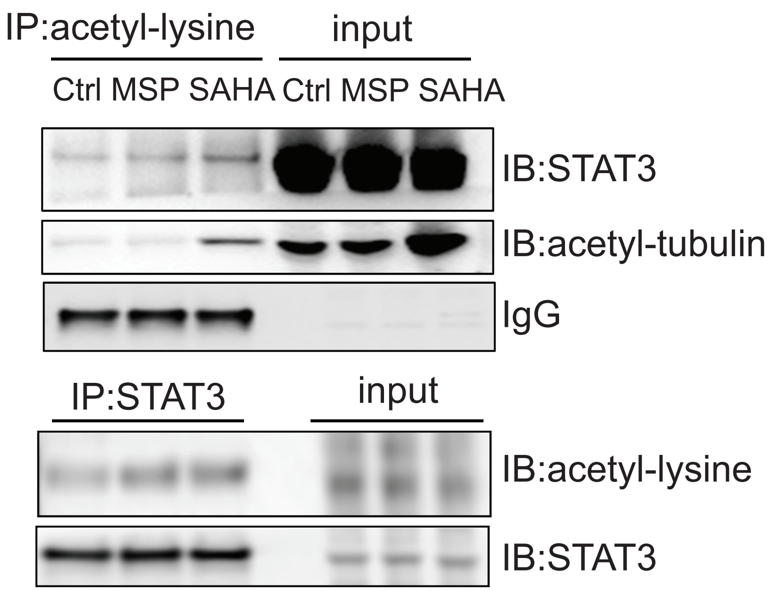

b

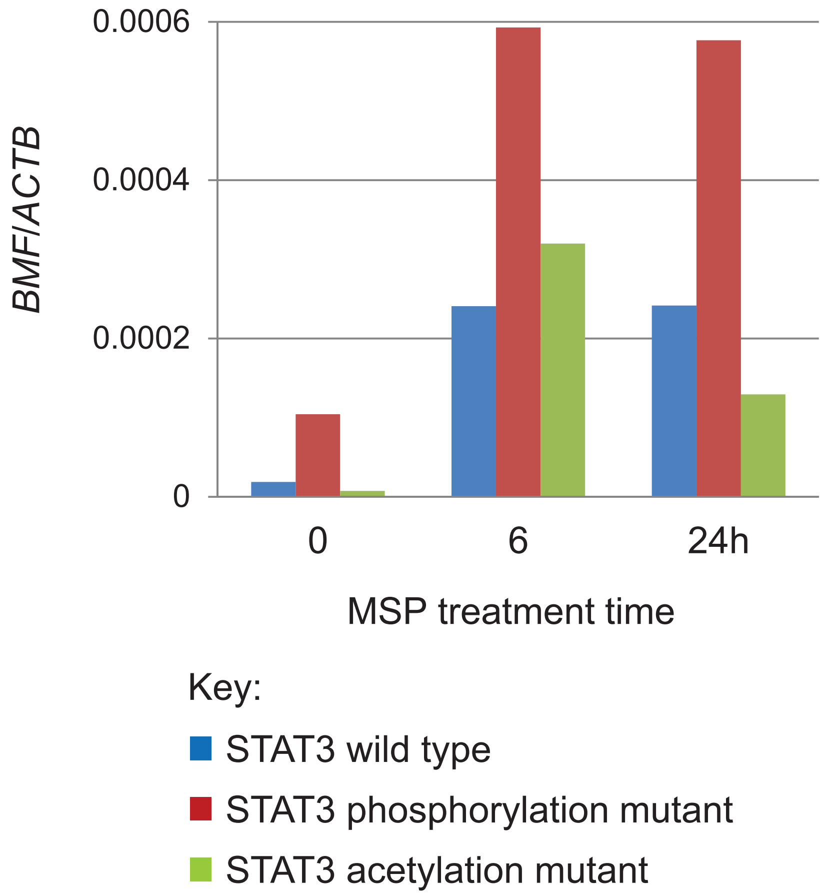

Supplementary Fig 9
